# Problematic mobile phone use and time management disposition in Chinese college students: the chain mediating role of sleep quality and cognitive flexibility

**DOI:** 10.1186/s40359-023-01481-z

**Published:** 2023-12-13

**Authors:** Yidan Yuan, Xinyue He, Quanxing He, Yimeng Jia, Zhansheng Xu, Man Li

**Affiliations:** 1 Faculty of Psychology, Tianjin Normal University, Tianjin, 300387 China; 2 Key Research Base of Humanities and Social Sciences of the Ministry of Education, Academy of Psychology and Behavior, Tianjin Normal University, Tianjin, 300387 China; 3 Tianjin Social Science Laboratory of Students’ Mental Development and Learning, Tianjin, 300387 China

**Keywords:** Time management disposition (TMD), Problematic mobile phone use (PMPU), Sleep quality, Cognitive flexibility

## Abstract

**Background:**

With the widespread adoption of smartphones, there has been a notable increase in problematic mobile phone use (PMPU), particularly prevalent among college students. Research suggests that apart from being associated with various problematic behaviors, this excessive mobile phone use might also have an impact on individual personality traits, such as time management disposition (TMD), which plays a significant role in individual motivation and psychological well-being. While previous literature has identified a negative relationship between PMPU and TMD, few studies have delved into the underlying mediating mechanism. Thus, the main aim of this study was to examine the chain mediating effect of sleep quality and cognitive flexibility on the relationship between PMPU and TMD.

**Methods:**

A total of 921 Chinese college students completed the questionnaire. We collected basic information about the participants and assessed their PMPU, TMD, sleep quality, and cognitive flexibility using the Problematic Mobile Phone Use Scale-10, Adolescence Time Management Disposition Inventory, Pittsburgh Sleep Quality Index scale and Cognitive Flexibility Inventory.

**Results:**

The results indicated a significant correlation among all the variables. Moreover, we noted that both sleep quality and cognitive flexibility fully mediated the association between PMPU and TMD. Additionally, a chain mediating effect involving sleep quality and flexibility in this relationship was also identified.

**Conclusion:**

We found that sleep quality and cognitive flexibility had a series of multiple mediating effects in the pathway from PMPU to TMD, and both significantly mediated TMD. These findings indicated that impaired cognitive function and sleep quality may contribute to time management difficulties resulting from PMPU, suggesting that problematic behaviors like PMPU can impact one’s personality traits. Therefore, interventions should be enhanced to mitigate the adverse effects of PMPU.

## Introduction

Time management disposition (TMD) reflects how individuals use and control their time. It is a multi-dimensional personality trait that encompasses three dimensions: the sense of time value, the sense of time control, and the sense of time efficacy [1]. Its negative effect on college students’ academic performance, motivation, and mental health is gradually gaining attention [2–5]. These studies have focused on the effect of TMD and suggest that increased attention to TMD may help students improve their academic achievement and prevent adverse mental health outcomes. Few studies, however, have explored the predictors of TMD, which could help students improve time efficiency. Thus, it is important to identify the influencing factors of TMD and understand its underlying mechanisms in order to develop effective interventions.

### Problematic mobile phone use and time management disposition

Problematic mobile phone use (PMPU) is defined as a problematic online behavior characterized by an inability to regulate use of the Internet on smartphone [6, 7]. A large-scale study found that the prevalence of problematic mobile phone use was as high as 27.5% among Chinese college students [8]. Due to the heightened loneliness resulting from home quarantine and increased negative emotions during the COVID-19 pandemic, smartphone addiction has become a prominent issue among students in mainland China [9]. Extensive research substantiates that PMPU adversely affects individuals, leading to issues such as the difficulty concentrating, an increased risk of suicide, and depression [10, 11]. Therefore, it is imperative to investigate PMPU in college students to enhance their overall mental and physical well-being.

According to the temporal decision model [12], people will delay the task if the outcome utility of the task is weaker than the current task aversiveness. PMPU meets the need for passing time and entertainment, and helps people escape from pressure [13–15], thus may disturb the current assessment of task and result in time management failure [16]. Subsequent research has revealed that students with mobile phone dependence exhibit inadequate TMD, leading to more time management failure regarding academic [17]. This suggests a potential correlation between PMPU and impaired TMD. In detail, it can be inferred that individuals who engage excessively and problematically with mobile phones may experience difficulties in managing their time effectively [4]. This relationship, as well as its correlative mechanism, needs to be verified in further research.

### The potential mediating role of sleep quality

Previous studies have demonstrated that PMPU is a significant predictor of poor sleep quality [11, 18]. Excessive mobile phone use not only results in bedtime procrastination, but also leads to hyperarousal, which can impede falling asleep and reduce sleep quality [18–20]. For instance, a longitudinal study suggested that students with prolonged PMPU may experience a disturbed circadian rhythm, resulting in poor sleep quality [21]. This diminished sleep quality, in turn, could compromise individuals’ self-monitoring abilities, making it challenging to effectively manage their time [5, 22]. In essence, it can be inferred that individuals engaging in problematic mobile phone use may encounter impaired sleep quality, contributing to a higher likelihood of experiencing TMD. Recent research has indicated that both PMPU and time management are associated with sleep quality [23], while the link between PMPU and TMD remains underexplored. The present study, delving into potential relationships across these three variables, serves to bridge this knowledge gap.

### The potential mediating role of cognitive flexibility

Cognitive flexibility, which may explain the link between PMPU and TMD, is the ability to adjust one’s view and behavior to improve adaptability in a changing environment [24, 25]. An Interaction of Person-Affect-Cognition-Execution model (I-PACE) is a process model that underscores Internet-use disorder as the outcome of interactions among predisposing factors (such as personality), moderators (such as attentional biases), and mediators (such as executive function) [26]. Moreover, this model suggests that long-term Internet-use disorder could reinforce these processes by influencing its contributing factors. According to the I-PACE model, individuals with poorer cognitive flexibility are more likely to engage in PMPU, and prolonged PMPU, in turn, may impair their cognitive flexibility. Consequently, individuals exhibiting problematic mobile phone use may experience diminished cognitive flexibility, contributing to difficulties in time management, such as procrastination [17, 27]. It could be inferred that TMD, a personality trait regarding time management, could be influenced by PMPU through decreased cognitive flexibility.

### The potential chain mediating role of sleep quality and cognitive flexibility

The strong correlation between sleep quality and cognitive flexibility has been demonstrated by several studies. For example, individuals may experience impaired cognitive flexibility as a result of poor sleep quality [28]. Furthermore, research indicates that a single night of recovery sleep can mitigate this negative impact on cognitive flexibility [29]. These findings imply that compromised sleep quality disrupts the restoration of cognitive flexibility, leading to a decrease in cognitive flexibility. This relationship provides a third pathway in the model, wherein PMPU-induced poor sleep quality leads to diminished cognitive flexibility, subsequently contributing to a reduction in TMD due to an increase in time management failures [17, 18, 27].

### The current study

**Fig. 1:**
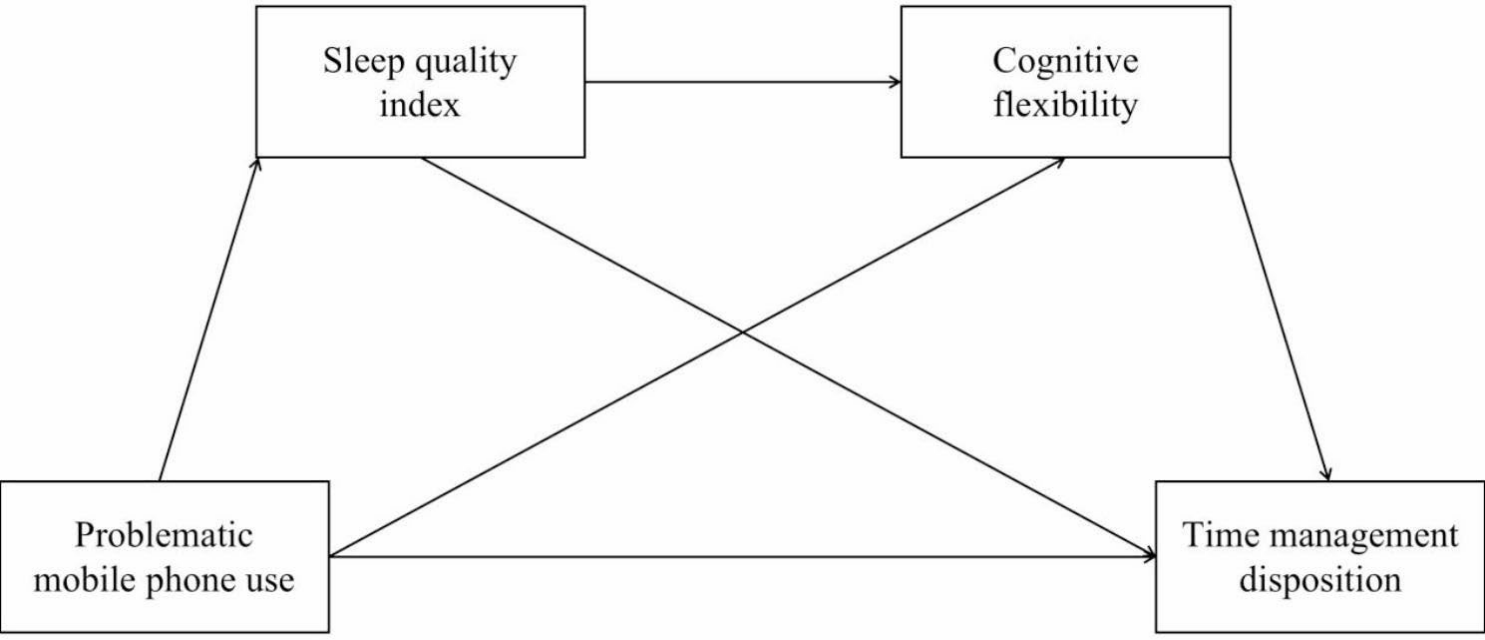
Hypothesized relationships between Problematic mobile phone use, sleep quality, cognitive flexibility and Time management disposition

Several adverse outcomes are related to PMPU [11], and a poorer TMD is one of them [17]. However, the mechanism underlying the association between PMPU and TMD has not been explored. To the best of our knowledge, this study represents the first systematic investigation into the potential mediating impact of sleep quality and cognitive flexibility on the relationship between PMPU and TMD. This insight establishes a foundation for interventions aimed at enhancing students’ time management skills. Furthermore, it contributes valuable evidence to the I-PACE model, substantiating the relationships among PMPU, cognitive flexibility, and TMD. Considering the correlations between sleep quality, cognitive flexibility and two main variables [28, 30, 31], the current study assumed that sleep quality and cognitive flexibility play the pivotal role in the relationship between PMPU and TMD. To validate this inference and address the knowledge gaps, a chain mediation model was examined which could demonstrate that PMPU is related to TMD through two associative variables, such as sleep quality and cognitive flexibility (shown in Fig. 1). Based on previous studies, four hypotheses were developed: (1) PMPU would negatively predict TMD; (2) PMPU would indirectly affect TMD through sleep quality; (3) PMPU could indirectly affect TMD through cognitive flexibility; and (4) both sleep quality and cognitive flexibility exhibit a chain mediating effect in the relationship between PMPU and TMD.

## Method

### Participants

Questionnaire data were collected using an online platform, with the support of teachers from a university in Tianjin Province, China. Full-time students received the questionnaire link from their teachers and were encouraged to share it with their peers. Each participant received information about the survey and was asked to sign the informed consent form. Initially, 1058 participants were recruited, but 137 were excluded due to incomplete questionnaires. The final sample size consisted of 921 participants (Mage = 19.78 years, SD = 1.74), comprising 656 (71.2%) males and 265 (28.8%) females. Regarding family socioeconomic status, 72.1% of participants had lived in an urban environment before the age of 18, and 59.2% of their parents had a junior or senior high school degree. Additionally, 61.5% of participants came from middle-income families whose monthly income ranged from RMB 5000 to 30,000 [32].

### Procedures

The design of this study was approved by the Institutional Review Board. Participants were surveyed online after signing the informed consent form, in which they were made aware that there were no right or wrong answers and that all personal information would remain confidential. Each participant completed the Mobile Phone Problem Use Scale (MPPUS-10), Pittsburgh Sleep Quality Index (PSQI), Cognitive Flexibility Inventory (CFI), and Adolescence Time Management Disposition Inventory (ATMDI).

### Measures

#### Problematic mobile phone use

We used the Chinese version of Problematic Mobile Phone Use Scale-10, which was translated from the short scale MPPUS-10 [33], to measure the PMPU of participants. The scale had demonstrated good reliability and validity in previous research [34]. The questionnaire consisted of 10 items, each scored on a scale from 1 to 5, with 1 indicating “strongly disagree” and 5 indicating “strongly agree”. The higher the score, the more severe the problematic mobile phone use. The Cronbach’s α in this study was 0.88.

#### Sleep quality

The Pittsburgh Sleep Quality Index was used to measure participants’ sleep quality in the last month [35]. The 18 self-reported items were grouped into 7 component scores (subjective sleep quality, sleep latency, sleep duration, habitual sleep efficiency, sleep disturbances, use of sleeping medications, and daytime dysfunction), and each was scored from 0 to 3. The sum of the 7 scores constituted the sleep quality index, with a higher score indicating worse sleep quality. The Chinese version had been validated in college students [36]. In this study, the Cronbach’s α was 0.71.

#### Cognitive flexibility

We assessed the participants’ cognitive flexibility using the Cognitive Flexibility Inventory [37], which had demonstrated good reliability and validity [38]. This scale consisted of 20 items, which were divided into two factors: alternatives and control. Respondents were asked to rate each item on a 5-point scale, with 1 indicating “never” and 5 indicating “always”. Thus, higher scores indicated greater cognitive flexibility, and the Cronbach’s α for this scale was 0.93.

#### Time management disposition

The Adolescence Time Management Disposition Inventory was used to measure TMD [39]. This scale contained 44 items, which were divided into 3 factors: the sense of time value, the sense of time control, and the sense of time efficacy. Each item was rated on a 5-point Likert scale, ranging from 1 (*strongly disagree*) to 5 (*strongly agree*). Higher scores indicated a higher level of TMD, and the Cronbach’s α for this scale was 0.96 in this study.

### Data analysis

Pearson correlation analysis was conducted using SPSS 26.0. Subsequently, Hayes’ [40]PROCESS program was employed to perform mediation analysis. Mediation models were examined using model 6 in PROCESS to test the hypotheses. A bias-corrected bootstrapping test with a 95% confidence interval (CI) from 5000 resamples was adopted to calculate the indirect effects; an indirect effect was deemed significant if the 95% CI did not contain 0. All values in the model were standardized, and gender and age were included as covariates.

Another chain mediation model was tested to compare with the hypothesized model in Fig. 1. In this model, TMD was examined as independent variable and PMPU as dependent variable.

## Results

### Common method bias test

Harman single-factor test was used to prevent common method bias [41]. Items from all questionnaires entered to the model and the first principal component explained 32.50% (lower than 40%) of the variance. Therefore, the common method bias did not influence the results in the current study.

### Descriptive analysis and correlation analysis

Descriptive data and bivariate correlation data were presented in Table 1. PMPU was positively correlated with sleep quality index (r = 0.37, *p* < 0.01) and negatively correlated with cognitive flexibility (r = -0.20, *p* < 0.01), as well as TMD (r = -0.28, *p* < 0.01). Sleep quality index was found to be negatively correlated with cognitive flexibility (r = -0.17, *p* < 0.01). Furthermore, TMD was negatively associated with sleep quality index (r = -0.26, *p* < 0.01) and positively correlated with cognitive flexibility (r = 0.62, *p* < 0.01). Additionally, age was negatively correlated with PMPU (r = -0.11, *p* < 0.01), and no other correlations were statistically significant.

**Table 1:** Demographic characteristics and correlations

	Mean	SD	1	2	3	4	5	6
1. Age	19.78	1.74	1					
2. Gender	0.71	0.45	-0.03	1				
3. PMPU	27.24	8.41	-0.11^**^	-0.05	1			
4. Sleep quality index	2.94	2.58	0.02	0.00	0.37^***^	1		
5. Cognitive flexibility	73.27	12.82	-0.02	0.02	-0.20^***^	-0.17^***^	1	
6. TMD	154.53	27.53	0.01	-0.05	-0.18^***^	-0.26^***^	0.62^***^	1

*Note.* Gender (0 = *female*, 1 = *male*); SD = standardized deviation; PMPU = problematic mobile phone use; TMD = time management disposition; a high sleep quality index indicated the poor sleep quality; ^**^p < 0.01; ^***^p < 0.001

### Chain mediation analysis

The results of the chain mediating effect of sleep quality index and cognitive flexibility were shown in Table 2. The chain mediating model showed satisfactory fit after controlling gender and age (χ2/df = 0, CFI = 1, TLI = 1, RMSEA = 0, SRMR = 0, *F*(5, 915) = 127.16, *R*^2^ = 0.41, *p* < 0.001). The result showed significant total effect of PMPU on TMD (Standardized *β* = -0.19, SE = 0.11, 95% CI [-0.80, -0.40]). The total indirect effect of PMPU on TMD was significant (indirect effect *=* -0.18, *p* < 0.05, 95% Bootstrap CI [-0.23, -0.13]), with an effect size (ratio of indirect to total effect) of 98.97%. Additionally, the direct effect in this model was not significant (Standardized *β* = -0.002, SE = 0.09, 95% CI [-0.19, 0.17]), as well as the effect of age (Standardized β = -0.02, SE = 0.52, 95% CI [-1.28, 0.75]) and gender (Standardized β = -0.06, SE = 1.97, 95% CI [-7.57, 0.18]) on TMD. Moreover, the model had three pathways. PMPU is associate with TMD through the full mediating effect of sleep quality index (indirect effect = -0.06, effect size = 32.99%, 95% Bootstrap CI = -0.08 to -0.04) and cognitive flexibility (indirect effect = -0.10, effect size = 53.54%, 95% Bootstrap CI [-0.15, -0.05]) respectively. PMPU is also related to TMD through the chain mediating effect of sleep quality index and cognitive flexibility (indirect effect = -0.02, effect size = 12.44%, 95% Bootstrap CI [-0.04, -0.01]).

The comparisons of indirect effects were also shown in Table 2. Comparison 1 indicated that the difference between the indirect effect of sleep quality index and cognitive flexibility was not significant (95% Bootstrap CI [-0.02, 0.10]). According to the other comparisons, the indirect effect of the chain mediating path was significantly different from that of sleep quality index (95% Bootstrap CI [-0.07, -0.01]) and cognitive flexibility (95% Bootstrap CI [-0.13, -0.02]).

**Table 2:** Result of chain mediation analysis

Standardized indirect effects	Indirect effect	Boot SE	Boot LLCI	Boot ULCI	Effect size
Total indirect effect	-0.18	0.03	-0.23	-0.13	98.97%
Path 1:					
PMPU → sleep quality index → TMD	-0.06	0.01	-0.08	-0.04	32.99%
Path 2:					
PMPU → cognitive flexibility → TMD	-0.10	0.03	-0.15	-0.05	53.54%
Path 3:					
PMPU → sleep quality index → cognitive flexibility → TMD	-0.02	0.01	-0.04	-0.01	12.44%
Comparison 1	0.04	0.03	-0.02	0.10	
Comparison 2	-0.04	0.02	-0.07	-0.01	
Comparison 3	-0.08	0.03	-0.13	-0.02	

*Note.* Three paths with indirect effect were shown in the table. Gender was coded 0 = *females*, 1 = *males*. PMPU = problematic mobile phone use; TMD = time management disposition; Boot SE = the standard error of 95% Bootstrap confidence interval of indirect effect; Boot LLCI = lower limits of 95% Bootstrap confidence interval of indirect effect; Boot ULCI = upper limits of 95% Bootstrap confidence interval of indirect effect; relative mediation effect = the ratio of indirect effect and total effect; Comparison 1: the indirect effect of path 1 minus that of path 2; Comparison 2: the indirect effect of path1 minus that of path3; Comparison 3: the indirect effect of path 2 minus that of path3

**Fig. 2:**
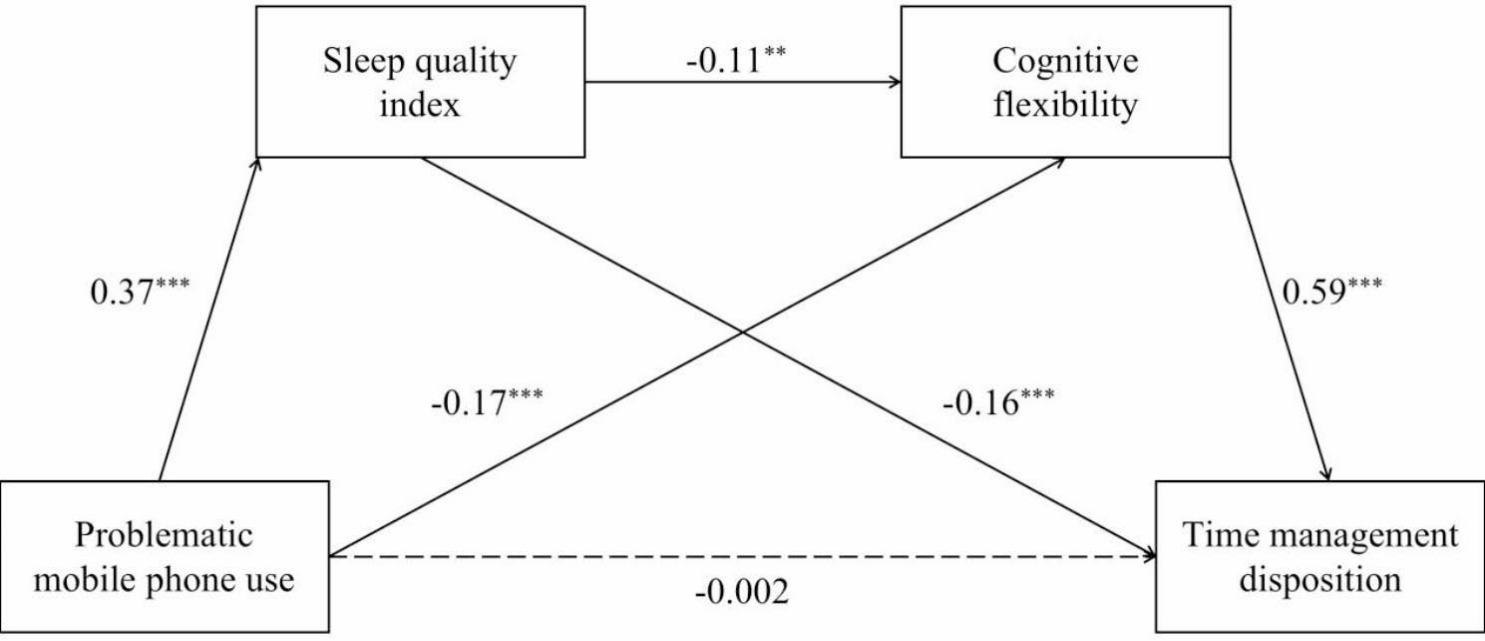
The chain mediating effect of sleep quality and cognitive flexibility between PMPU and TMD. *Note*. Path values were standardized coefficients. ^*^p < 0.05; ^**^p < 0.01; ^***^p < 0.001

These results indicated that PMPU could predict TMD through the chain mediation effect of sleep quality and cognitive flexibility (Fig. 2). Furthermore, both sleep quality and cognitive flexibility played a full mediating role in the association between PMPU and TMD.

**Fig. 3:**
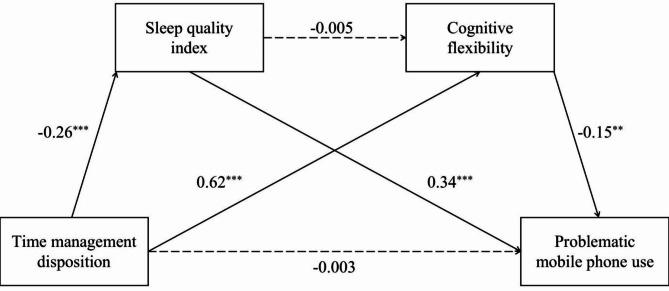
The chain mediating effect of sleep quality and cognitive flexibility between TMD and PMPU

In consideration of the I-PACE model, which suggests the existence of bidirectional relationships or vicious circles between PMPU and its influencing factors, we explored an alternative model treating TMD as an independent variable (Fig. 3). TMD could be related to PMPU through sleep quality and cognitive flexibility respectively, but the chain mediating effect was not significant. Meanwhile, this model exhibited a poorer fit in comparison to the hypothesized model (*F*(5, 915) = 38.17, *R*^2^ = 0.17, *p* < 0.001), thereby partially confirming the effectiveness of the hypothesized model.

## Discussion

This study investigated the relationship between problematic mobile phone use (PMPU) and time management difficulties (TMD) by examining sleep quality and cognitive flexibility as mediators. The reliability of the results in the current study is well-supported by the satisfactory fitting indices of the hypothesized model. The most important finding of this study was that PMPU was associated with TMD through an indirect pathway, with sleep quality and cognitive flexibility acting as chain mediators. This result suggests that sleep quality and cognitive flexibility play a critical role in the relationship between PMPU and TMD. These findings provided preliminary evidence for the potential mechanism underlying the association between PMPU and TMD.

The participants in current study exhibited a higher level of PMPU compared to a previous study conducted in Heilongjiang Province, China (mean of the sum score: 5.13 ± 1.53) [42]. In addition to the influence of COVID-19 [9], geographic factors may affect students’ PMPU. For instance, adults in municipalities reported a longer duration of mobile phone use in a previous study [43].

Consistent with previous research, college students with PMPU were found to have a low level of TMD [4]. Taking into account the adverse effect of PMPU on academics, our results might explain how PMPU impairs students’ academic performance [44]. For instance, students with PMPU may have a lower level of TMD, which could lead to decreased efficiency in learning-related time management. The findings related to the second model also revealed that a reduction in TMD could account for the emergence of PMPU. In essence, the predictors and PMPU formed a vicious cycle consistent with the I-PACE model. According to the I-PACE model, these vicious cycles extend across PMPU, affective and cognitive responses, and executive function [26]. The results of the current study suggest the possibility of a bidirectional process between PMPU and personality, thus expanding the model.

Our findings suggested that PMPU was associated with poor sleep quality among college students, which is consistent with prior research suggesting that disturbed circadian rhythms, such as melatonin rhythms, may play a role in this association [45, 46]. Moreover, our results revealed that poor sleep quality caused by PMPU was related to worse TMD which is in line with a previous study showing that better sleep quality was correlated to higher levels of TMD [47]. Time management difficulty has been considered in some literature as an aspect of executive function impairment, potentially induced by poor sleep quality [28, 48]. In essence, PMPU may disrupt circadian rhythms, subsequently leading to diminished sleep quality. This compromised sleep quality, in turn, can impair time management, which relies on executive function. Consequently, PMPU exerts a long-term effect on TMD.

This study also found that college students with a higher level of PMPU would perform worse in cognitive flexibility. Thornton [49] demonstrated that cognitive flexibility could be adversely affected by the mere presence of a mobile phone. This suggests that mobile phones may act as a distraction, reducing the available cognitive resources, particularly for individuals with PMPU. In other words, prolonged use of mobile phones can automatically occupy individuals’ cognitive resources, impairing their cognitive flexibility. This poor cognitive flexibility, in turn, correlates with increased time management failure, indicative of worsened TMD [17, 50]. As a result, PMPU may lead to a depletion of cognitive resources available for effective time management, ultimately contributing to diminished TMD.

The core finding of this study was the chain-mediating effect of cognitive flexibility and sleep quality between PMPU and TMD. This result was consisted with previous study in which PMPU was linked to poor executive function through a decline in sleep quality [51]. Individuals who excessively used mobile phones tended to experience poor sleep quality, attributed to disrupted circadian rhythms [45, 46]. Taking into account the protective influence of sleep quality on cognitive flexibility [52], this impaired sleep quality may then interfere with the recovery of cognitive resources, which increased the failure of time management and, consequently, reduced TMD.

The current study had several limitations. Firstly, all variables were self-reported, which could lead to discrepancies in results due to participants’ subjective interpretations. To address this, further studies should consider using more objective measurements, such as cognitive flexibility tests or physiological indices for sleep quality. Additionally, there was a disproportionate number of males to females in the study. Though there were no significant correlations between gender and other variables in this study, future studies should take gender into account when recruiting participants. Furthermore, participants in the current study were recruited exclusively from one university, thereby partially limiting the representativeness of the sample. Hence, future studies should consider recruiting subjects from multiple universities or provinces. Finally, the cross-sectional design limited the ability to draw causative conclusions about the relationships between variables in this study. To better understand these relationships, longitudinal research is needed to determine if PMPU and TMD are predictors or outcomes.

Despite these limitations, this study was the first to examine the mediating role of sleep quality and cognitive flexibility in the relationship between PMPU and TMD in college students. It provided an insight into the mechanism underlying this association. Moreover, previous studies have shown that PMPU can lead to adverse outcomes such as behavioral problems [2]. This study, however, was the first to focus on the personality trait of time management. It suggested that high levels of PMPU can lead to decreased TMD and inefficiency in time management in college students. Additionally, this study helped to fill a gap in the knowledge of the predicting factors of TMD, and established a framework for intervening to prevent the adverse consequences of PMPU. For instance, a study revealed improvements in individuals’ sleep quality and working memory following restrictions on mobile phone use before bedtime [53].

## Conclusion

This study verified the mediating role of the chain relationship between sleep quality and cognitive flexibility in the association between PMPU and TMD. Notably, PMPU was only associates with TMD through sleep quality and cognitive flexibility, and not directly. The results showed that PMPU led to a decrease in sleep quality, resulting in individuals having fewer cognitive resources for time management. Thus, individuals with PMPU should be made aware of the importance of addressing their sleep quality and cognitive flexibility. Additionally, the findings highlighted the crucial roles of sleep quality and cognitive flexibility in time management training.

## Data Availability

The datasets analysed during the current study are not publicly available but are available from the corresponding author on reasonable request.

## Abbreviations

TMD: Time management disposition
PMPU: Problematic mobile phone use
I-PACE: Person-Affect-Cognition-Execution model
MPPUS-10: Mobile Phone Problem Use Scale
PSQI: Pittsburgh Sleep Quality Index
CFI: Cognitive Flexibility Inventory
ATMDI: Adolescence Time Management Disposition Inventory
CI: Confidence interval
SD: Standardized deviation
Boot SE: The standard error of 95% Bootstrap confidence interval
Boot LLCI: Lower limits of 95% Bootstrap confidence interval
Boot ULCI: Upper limits of 95% Bootstrap confidence interval
